# High-Frequency 10-kHz Spinal Cord Stimulation Improves Health-Related Quality of Life in Patients With Refractory Painful Diabetic Neuropathy: 12-Month Results From a Randomized Controlled Trial

**DOI:** 10.1016/j.mayocpiqo.2022.05.003

**Published:** 2022-07-01

**Authors:** Erika A. Petersen, Thomas G. Stauss, James A. Scowcroft, Elizabeth S. Brooks, Judith L. White, Shawn M. Sills, Kasra Amirdelfan, Maged N. Guirguis, Jijun Xu, Cong Yu, Ali Nairizi, Denis G. Patterson, Kostandinos C. Tsoulfas, Michael J. Creamer, Vincent Galan, Richard H. Bundschu, Neel D. Mehta, Dawood Sayed, Shivanand P. Lad, David J. DiBenedetto, Khalid A. Sethi, Johnathan H. Goree, Matthew T. Bennett, Nathan J. Harrison, Atef F. Israel, Paul Chang, Paul W. Wu, Charles E. Argoff, Christian E. Nasr, Rod S. Taylor, David L. Caraway, Nagy A. Mekhail

**Affiliations:** a Department of Neurosurgery, University of Arkansas for Medical Sciences, Little Rock; b Department of Anesthesiology, University of Arkansas for Medical Sciences, Little Rock; c Advanced Pain Management, Greenfield, WI; d Pain Management Associates, Lee’s Summit, MO; e Nevro Corp, Redwood City, CA; f AES Compass Orlando, Orlando, FL; g Touchstone Interventional Pain Center, Medford, OR; h IPM Medical Group, Walnut Creek, CA; i Ochsner Health System, New Orleans, LA; j Department of Pain Management, Cleveland Clinic Foundation, Cleveland, OH; k Department of Endocrinology, Cleveland Clinic Foundation, Cleveland, OH; l Swedish Medical Center, Seattle, WA; m Nevada Advanced Pain Specialists, Reno, NV; n Central Florida Pain Relief Centers, Orlando, FL; o Pain Care, Stockbridge, GA; p Coastal Orthopedics and Sports Medicine, Bradenton, FL; q Department of Anesthesiology, Weill Cornell Medical College, New York, NY; r Department of Anesthesiology and Pain Medicine, University of Kansas Medical Center, Kansas City, KS; s Department of Neurosurgery, Duke University, Durham, NC; t Boston PainCare, Waltham, MA; u Department of Neurosurgery, United Health Services, Johnson City, NY; v Holy Cross Hospital, Fort Lauderdale, FL; w Department of Neurology, Albany Medical Center, Albany, NY; x MRC/CSO Social and Public Health Sciences Unit & Robertson Centre for Biostatistics, Institute of Health and Well Being, University of Glasgow, Glasgow, United Kingdom

**Keywords:** CMM, conventional medical management, DN4, Douleur Neuropathique, DSPN, diabetic sensorimotor peripheral neuropathy, EQ-5D-5L, EuroQol 5-Dimension 5-Level questionnaire, HbA1c, hemoglobin A1c, HRQoL, health-related quality of life, IPG, implantable pulse generator, NNT, number needed to treat, PDN, painful diabetic neuropathy, RCT, randomized controlled trial, SCS, spinal cord stimulation, VAS, visual analog scale

## Abstract

**Objective:**

To evaluate high-frequency (10-kHz) spinal cord stimulation (SCS) treatment in refractory painful diabetic neuropathy.

**Patients and Methods:**

A prospective, multicenter randomized controlled trial was conducted between Aug 28, 2017 and March 16, 2021, comparing conventional medical management (CMM) with 10-kHz SCS+CMM. The participants had hemoglobin A1c level of less than or equal to 10% and pain greater than or equal to 5 of 10 cm on visual analog scale, with painful diabetic neuropathy symptoms 12 months or more refractory to gabapentinoids and at least 1 other analgesic class. Assessments included measures of pain, neurologic function, and health-related quality of life (HRQoL) over 12 months with optional crossover at 6 months.

**Results:**

The participants were randomized 1:1 to CMM (n=103) or 10-kHz SCS+CMM (n=113). At 6 months, 77 of 95 (81%) CMM group participants opted for crossover, whereas none of the 10-kHz SCS group participants did so. At 12 months, the mean pain relief from baseline among participants implanted with 10-kHz SCS was 74.3% (95% CI, 70.1-78.5), and 121 of 142 (85%) participants were treatment responders (≥50% pain relief). Treatment with 10-kHz SCS improved HRQoL, including a mean improvement in the EuroQol 5-dimensional questionnaire index score of 0.136 (95% CI, 0.104-0.169). The participants also reported significantly less pain interference with sleep, mood, and daily activities. At 12 months, 131 of 142 (92%) participants were “satisfied” or “very satisfied” with the 10-kHz SCS treatment.

**Conclusion:**

The 10-kHz SCS treatment resulted in substantial pain relief and improvement in overall HRQoL 2.5- to 4.5-fold higher than the minimal clinically important difference. The outcomes were durable over 12 months and support 10-kHz SCS treatment in patients with refractory painful diabetic neuropathy.

**Trial registration:**

clincaltrials.gov Identifier: NCT03228420

The World Health Organization estimates 422 million individuals with diabetes worldwide, with projections reaching 700 million by 2045.^1^^,^^2^ Neuropathies comprise the most common complication of diabetes, and diabetic sensorimotor peripheral neuropathy (DSPN) is the most prevalent type.^3^ The symptoms of DSPN, including numbness, tingling, and frequently neuropathic pain, appear initially in the toes and feet bilaterally and migrate proximally. Disease progression may manifest upper limb symptoms affecting the fingers and hands and ascending the arms, resulting in a “stocking and glove” distribution classic for DSPN.

Diabetic sensorimotor peripheral neuropathy can be debilitating; numbness increases the risk of falling and causes loss of protective sensation associated with foot ulceration and increased mortality.^4^^,^^5^ Nearly half of the patients with DSPN develop painful diabetic neuropathy (PDN) causing extremity pain, paresthesia, burning, and shooting pain that is typically worse at night and disrupts sleep.^6^^,^^7^ Painful diabetic neuropathy therapies target pain management and enhanced glycemic control to mitigate further nerve damage as there are currently no disease-modifying treatments.^3^^,^^8^, ^9^, ^10^

Several neuropathic pain medications are recommended.^8^^,^^11^ Typical first-line agents include gabapentinoids and serotonin-norepinephrine reuptake inhibitors. Tricyclic antidepressants are commonly prescribed. Patients with inadequate pain relief may be prescribed opioids. A meta-analysis of randomized controlled trials (RCTs) on neuropathic pain medication found that the number needed to treat (NNT) for 50% pain reduction ranged from 3.6 to 10.6.^12^ Insufficient pain relief or adverse effects lead to poor long-term medication adherence, with 25% to 35% of patients discontinuing after 1 month and 65% to 75% discontinuing over 1 year.^13^^,^^14^

Spinal cord stimulation (SCS) modulates chronic pain pathways with mild electrical pulses and has been evaluated for PDN.^15^, ^16^, ^17^, ^18^, ^19^, ^20^ The traditional SCS approach applies low-frequency (40-60 Hz) pulses that generate paresthesia, and treatment success requires overlapping this paresthesia with the painful area.^21^ A more recent approach applying high-frequency (10 kHz) pulses preferentially activates inhibitory dorsal horn interneurons, does not produce paresthesia, and provides superior pain relief for chronic back and leg pain.^22^, ^23^, ^24^, ^25^ Recent data suggest that 10-kHz SCS also provide substantial pain relief and improve sensation in patients with PDN.^26^, ^27^, ^28^, ^29^

Current PDN treatment options remain inadequate for many patients, resulting in large unmet needs. Investigations with low-frequency SCS report modest pain relief; however, this treatment has not been widely adopted in clinical practice. This study assesses long-term effects of high-frequency 10-kHz SCS on pain, neurologic function, and health-related quality of life (HRQoL) in patients with PDN with refractory symptoms.

## Patients and Methods

Methods have been described previously.^27^^,^^28^^,^^30^ This study was designed to be a pragmatic clinical trial^31^ with 18 US sites. The patients recruited reflect typical clinical practice as do the interventions used in the comparator arm. The protocol and informed consent were approved by the Western Institutional Review Board and appropriate local institutional review boards. The research staff conducted the study according to Good Clinical Practice and the Declaration of Helsinki. A portion of the study follow-up coincided with the COVID-19 pandemic. The investigators worked with institutional review boards on mitigation measures maximizing patient safety, including completing visits by telehealth and mail. Accordingly, some follow-up visits or in-person assessments could not be completed.

### Key Inclusion Criteria

Participants were aged 22 years or older with PDN symptoms for at least 12 months, currently or previously taking pregabalin or gabapentin plus at least 1 other class of analgesics; lower limb pain visual analog scale (VAS) of greater than or equal to 5 of 10 cm; hemoglobin A1c (HbA1c) level of less than or equal to 10%; and body mass index (BMI; calculated as the weight in kg divided by the height in m^2^) of less than or equal to 45 kg/m^2^. Investigators assessed comorbidities to determine participant suitability for SCS procedures.

### Randomization and Follow-up

Randomization 1:1 to conventional medical management (CMM) or 10-kHz SCS+CMM was completed at sites by blocks with concealed assignment. The participants were stratified by glycemic control (HbA1c level of <7% or ≥7%) and pain severity (VAS <7.5 cm or ≥7.5 cm). Assessments were completed at baseline and at 1, 3, 6, 9, and 12 months. The participants were eligible for crossover at 6 months if they had less than 50% pain relief from baseline, were dissatisfied with treatment, and the investigator approved it as medically appropriate.

### Outcome Measures

Lower limb pain was assessed on a 10-cm VAS.^32^ Other measures of pain included Douleur Neuropathique (DN4), the Short-form McGill Pain Questionnaire-2, and the Brief Pain Inventory for Diabetic Peripheral Neuropathy.^33^, ^34^, ^35^ Health-related quality of life was assessed with the EuroQol 5-Dimension 5-Level (EQ-5D-5L) and Diabetes Quality of Life questionnaires.^36^^,^^37^ Additionally, the impact of pain on sleep was assessed by the Pain and Sleep Questionnaire 3-Item Index.^38^ Clinicians rated participants’ mental well-being with Global Assessment of Functioning and completed the Clinician Global Impression of Change.^39^ The participants completed the Patient Global Impression of Change and satisfaction questionnaire. HbA1c levels were determined using standard blood tests.

A thorough neurologic examination was designed by 2 independent neurologists who trained the investigators to assess the lower limb motor strength, reflexes, light touch, and pinprick and 10-g monofilament sensation at 10 test sites per foot with Neuropen (Owen Mumford).^40^ The examination was consistent with the American Diabetes Association’s recommendations for assessing the loss of protective sensation and the American Academy of Neurology’s definition for diagnosing DSPN.^4^^,^^41^

### Conventional Medical Management

Patients continued their current therapy, and further optimization was permitted. The treatments included the usual noninvasive or minimally invasive therapies.^9^

### 10-kHz SCS

Temporary trial SCS lasting 1 to 2 weeks evaluated whether a participant obtained greater than or equal to 50% pain relief to be eligible for permanent implantation of the 10-kHz SCS system (Nevro Corp). During trial stimulation, 2 octopolar percutaneous leads were inserted into the thoracolumbar epidural space and connected to an external pulse generator. For permanent device implant, 2 leads were placed into the epidural space with electrodes spanning T8 to T11 vertebral bodies and connected to an implanted pulse generator (IPG). Stimulation was delivered at a 10-kHz frequency and 30-μs pulse width, with amplitude typically ranging from 0.5 to 4.0 mA.

### Statistical Analyses

Randomized participants who completed the 3-month primary end point visit were included in the per-protocol population for analysis. Those who did not complete the primary end point assessment remained in the study but were included only in the safety outcomes.

For continuous variables, descriptive statistics are reported. Paired *t* tests assessed the significance of the mean percentage change from baseline to 12 months within the treatment groups. For categorical variables, the numbers in each category are presented by the treatment groups and compared with the distributions across categories using the Fisher exact test.

Two-sided *P* values were considered significant at α level of 0.05. Given the exploratory and hypothesis generating nature of the analyses, *P* values were not adjusted for multiplicity. Statistical analyses were carried out using SAS version 9.4.

## Results

### Participants

**Figure 1:**
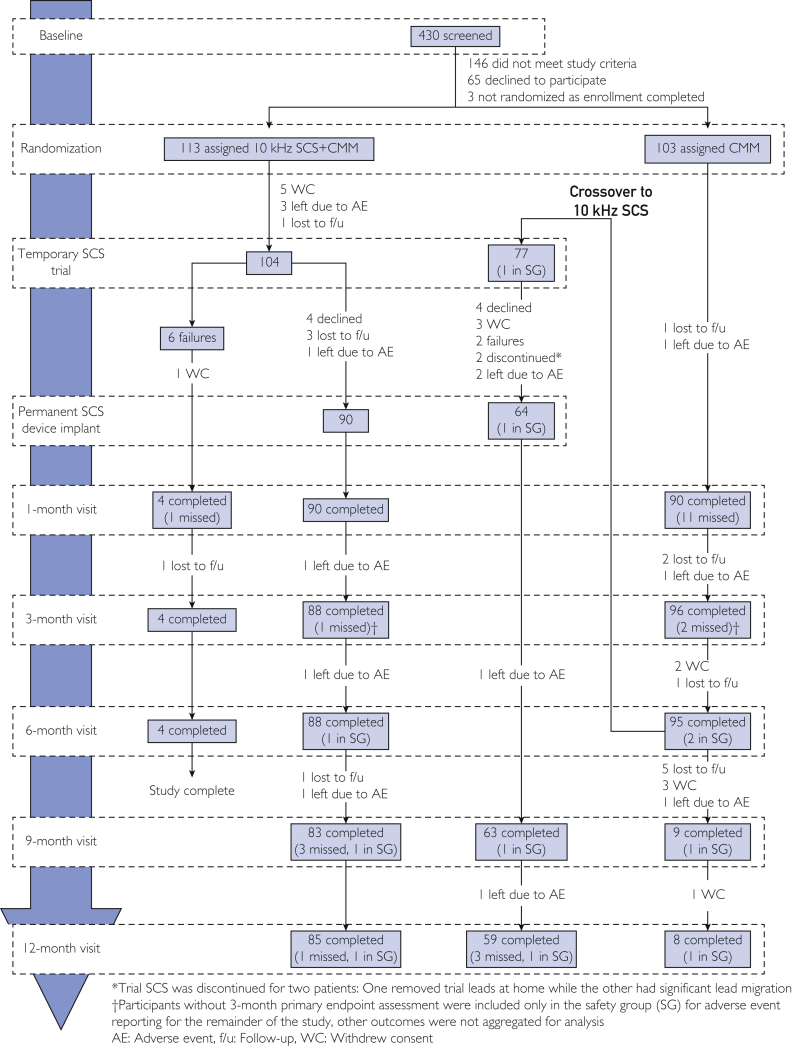
Disposition of all participants through 12 months. ^∗^Trial SCS was discontinued for 2 patients: 1 patient removed trial leads at home, whereas the other patient had a significant lead migration. ^†^Participants without the 3-month primary end point assessment were included only in the SG for AE reporting for the remainder of the study; other outcomes were not aggregated for analysis. AE, adverse event; CMM, conventional medical management; f/u, follow-up; SCS, spinal cord stimulation; SG, safety group; WC, withdrew consent.

In total, 103 participants were assigned to continue CMM and 113 were assigned the addition of 10-kHz SCS to CMM (Figure 1). The participants had a median duration of 10.9 years (interquartile range, 6.3-16.4) with diabetes and 5.6 years (interquartile range, 3.0-10.1) with painful neuropathy symptoms. Pain was moderate to severe, with mean lower limb VAS of 7.3 cm (SD, 1.6) and primarily neuropathic for 207 of 216 (96%) participants as reported by baseline DN4 score greater than or equal to 4, consistent with clinical PDN diagnosis.^42^ The mean age was 60.8 years (SD, 10.7), and 80 of 216 (37%) participants were women. Most participants (205 of 216, 95%) had type 2 diabetes, whereas the remainder had type 1. The mean HbA1c level was 7.4% (SD, 1.2), and the mean BMI was 33.7 kg/m^2^ (SD, 5.3).

### Analysis Groups

Temporary trial SCS was completed for 104 participants assigned to 10-kHz SCS+CMM. In the group that was assigned CMM, there were 95 who completed the 6-month follow-up visit and 81% (77 of 95) crossed from CMM to 10-kHz SCS+CMM, compared with none from the 10-kHz SCS+CMM arm (*P*<.001). Baseline characteristics for the resulting 3 analysis groups present at 12 months are reported in Table 1. Outcomes for participants who continued CMM or were excluded from per-protocol analysis are reported in Supplemental Figure 1 and Supplemental Tables 1 and 2 (available online at http://www.mcpiqojournal.org).

**Table 1:** Baseline Characteristics

Characteristic	10-kHz SCS+CMM n=104	CMM crossover to 10-kHz SCS n=77	CMM continuers n=18
Age (y), mean ± SD	60.5±11.3	60.3±10.1	62.4±10.3
Female, n (%)	38 (37%)	27 (35%)	6 (33%)
Male, n (%)	66 (63%)	50 (65%)	12 (67%)
Race			
White, n (%)	81 (77.9%)	66 (85.7%)	13 (72.2%)
Black or African American, n (%)	15 (14.4%)	8 (10.4%)	3 (16.7%)
Native Hawaiian or other Pacific Islander, n (%)	3 (2.9%)	1 (1.3%)	0
American Indian or Alaska Native, n (%)	2 (1.9%)	0	0
Asian, n (%)	1 (1.0%)	1 (1.3%)	0
Other, n (%)	2 (1.9%)	1 (1.3%)	2 (11.1%)
Diabetes			
Type 1, n (%)	8 (8%)	3 (4%)	0
Type 2, n (%)	96 (92%)	74 (96%)	18 (100%)
Duration (y)			
Diabetes, mean ± SD	13.1±8.5	12.5±9.1	11.7±6.8
Peripheral neuropathy, mean ± SD	7.5±5.8	6.8±4.9	7.6±5.9
HbA1c, mean ± SD	7.3%±1.1%	7.5%±1.2%	7.2%±1.0%
<7.0%, n (%)	40 (38%)	30 (39%)	7 (39%)
<8.0%, n (%)	73 (70%)	53 (69%)	15 (83%)
BMI (kg/m^2^), mean ± SD	33.7±5.3	34.0±5.3	34.1±5.0
Lower limb pain VAS (cm), mean ± SD	7.6±1.6	7.2±1.6	6.1±1.3
<7.5 cm, n (%)	48 (46%)	38 (49%)	17 (94%)
≥7.5 cm, n (%)	56 (54%)	39 (51%)	1 (6%)
Severity of neuropathic pain			
DN4, mean ± SD	6.6±1.7	6.4±2.0	6.6±1.9
<4, n (%)	2 (2%)	5 (7%)	1 (6%)
≥4, n (%)	102 (98%)	71 (93%)	17 (94%)

BMI, body mass index; CMM, conventional medical management; DN4, Douleur Neuropathique; HbA1c, hemoglobin A1c; SCS, spinal cord stimulation; VAS, visual analog scale.

### Pain Relief and Neurologic Function

Temporary trial SCS resulted in 171 of 179 (96%) participants having success, defined as at least 50% pain relief from baseline, and being eligible for permanent device implant. Among the original 10-kHz SCS+CMM group and those who crossed over from CMM, 154 participants proceeded with permanent implant. The full subject disposition is shown in Figure 1.

**Figure 2:**
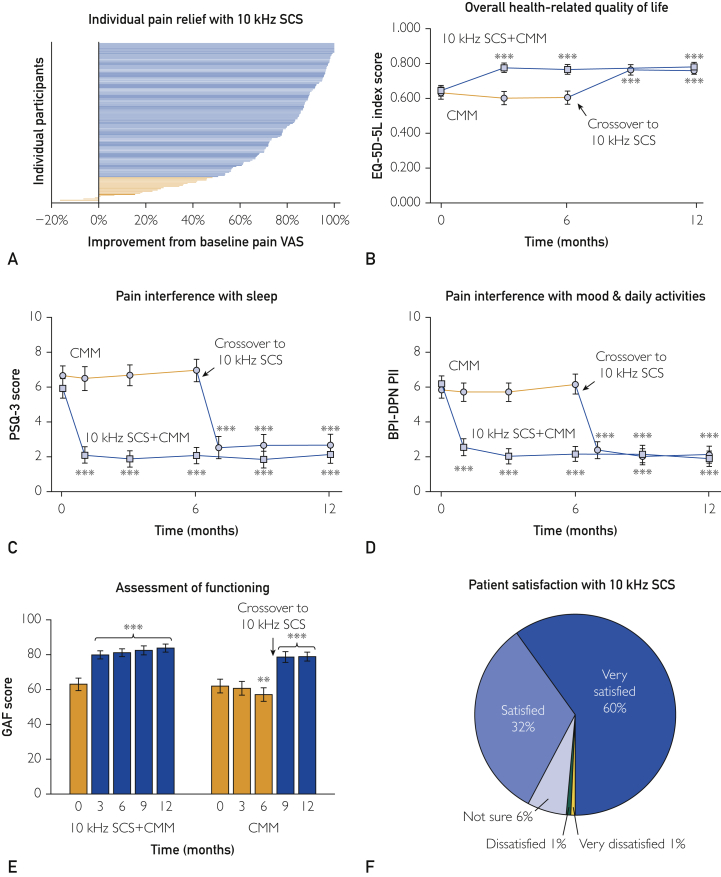
A, Individual pain responses among all 10-kHz SCS implanted participants (n=142) at 12 months. Each line represents the improvement in a single participant’s lower limb pain score relative to the baseline. Treatment responders (n=121) are those with at least 50% pain relief, shown in blue, with nonresponders (n=21) shown in orange. The average pain relief among all implanted participants at 12 months was 74%. B, The 5-level EQ-5D-5L measures health-related quality of life. Participants rate 5 items each with 5 levels of response that are converted to an index value on the basis of the US population norms, ranging from −0.109 (a state worse than death) to 1.000 (perfect health). The average scores are plotted over time for the 10-kHz SCS+CMM group participants (n=84, blue line, open squares) and CMM group participants who crossover after 6 months (n=57, orange line preimplant, blue line postimplant, open circles). C, The PSQ-3 evaluates the impact of pain on falling asleep and staying asleep with three 10-cm VASs, where 0 cm means “never” and 10 cm means “always.” The average scores are plotted over time for the 10-kHz SCS+CMM group participants (n=84, blue line, open squares) and CMM group participants who crossover after 6 months (n=58, orange line preimplant, blue line postimplant, open circles). D, The BPI-DPN scale has been validated in patients with PDN and assesses pain interference with activity, mood, and activities of daily living via 7 items on a scale of 0 (does not interfere) to 10 (completely interferes). The average scores are plotted over time for the 10-kHz SCS+CMM group participants (n=84, blue line, open squares) and CMM group participants who crossover after 6 months (n=57, orange line preimplant, blue line postimplant, open circles). E, The GAF scale requires clinician assessment of the patient’s mental health and well-being on a scale of 1 (persistent danger to oneself or others OR unable to maintain personal hygiene OR serious suicidality) to 100 (superior functioning in a wide range of activities/no symptoms). The average scores are shown over time for the 10-kHz SCS+CMM group participants (n=80, orange bar preimplant, blue bars postimplant) and CMM group participants who crossover after 6 months (n=56, orange bars preimplant, blue bars postimplant). F, The participants rated their satisfaction with treatment on a 5-point Likert scale ranging from “very dissatisfied” to “very satisfied” at 12 months. Proportions are shown for all implanted participants (n=142). Error bars: 95% CI, ∗*P*<.05, ∗∗*P*<.01, ∗∗∗*P*<.001. BPI-DPN, Brief Pain Inventory for Painful Diabetic Peripheral Neuropathy; CMM, conventional medical management; EQ-5D-5L, EuroQol 5-dimensional questionnaire; GAF, Global Assessment of Functioning; PDN, painful diabetic neuropathy; PSQ-3, Pain and Sleep Questionnaire 3-Item Index; SCS, spinal cord stimulation; VAS, visual analog scale.

The mean lower limb pain relief among all 10-kHz SCS implanted participants at 12 months was 5.6 cm (on 10-cm VAS) or 74.3% (95% CI, 70.1-78.5; Figure 2A). There were 121 of 142 (85%) treatment responders, defined as those with at least 50% pain relief from baseline. Further, 10-kHz SCS resulted in significant improvement in DN4 scores (*P*<.001) and across all Short-form McGill Pain Questionnaire-2 subscales (*P*<.05 for all subscales) (Table 2).

**Table 2:** Outcomes in the Per-Protocol Population Over 12 Months^a^^,^^b^

Outcome	10-kHz SCS+CMM			CMM Crossover to 10-kHz SCS after 6 mos		
	Baseline	6 mos	12 mos	Baseline	6 mos	12 mos
HbA1c, n		70			51	
mean ± SD	7.5%±1.2%	7.6%±1.4%	7.3%±1.2%	7.5%±1.1%	7.6%±1.3%	7.6%±1.2%
Lower limb pain, n		84			58	
10-cm pain VAS score (cm), mean ± SD	7.6±1.6	1.7±1.9^c^	1.7±1.8^c^	7.2±1.6	7.4±1.6	2.0±1.6^c^
Percent change from baseline, mean ± SD	-	−76%±26%^c^	−77%±25%^c^	-	7%±32%	−70%±27%^c^
Proportion of responders (%)	-	86%	86%	-	0	84%
DN4, n		77			52	
Total score, mean ± SD	6.6±1.8	3.4±2.4^c^	3.5±2.3^c^	6.6±2.0	6.7±2.1	3.5±2.4^c^
Proportion with score ≥4 (%)	97%	51%^c^	51%^c^	96%	92%	46%^c^
Proportion with score <4 (%)	3%	49%^c^	49%^c^	4%	8%	54%^c^
SF-MPQ-2, n		84			58	
Continuous pain, mean ± SD	5.2±2.5	1.6±1.7^c^	1.6±2.0^c^	5.4±2.6	5.7±2.4	1.7±1.6^c^
Intermittent pain, mean ± SD	5.4±2.5	1.7±2.1^c^	1.5±1.9^c^	5.6±2.2	6.0±2.5^d^	1.4±1.6^c^
Neuropathic pain, mean ± SD	5.5±2.0	1.9±1.6^c^	1.9±1.7^c^	5.4±2.1	5.7±2.1^d^	1.9±1.5^c^
Affective descriptors, mean ± SD	4.0±2.7	1.1±1.6^c^	1.0±1.7^c^	3.6±2.7	4.6±2.7^d^	0.7±1.1^c^
Total, mean ± SD	5.1±2.0	1.6±1.6^c^	1.6±1.7^c^	5.1±2.0	5.6±2.1^e^	1.5±1.3^c^
Neurological assessment, n		76			52	
Proportion with overall improvement (%)	-	59%	68%	-	0%	62%
Proportion with sensory improvement (%)	-	56%	66%	-	4%	58%
EQ-5D-5L, n		84			57	
Overall health VAS, mean ± SD	58.7±18.7	73.3±16.1^c^	75.6±18.6^c^	58.1±21.1	56.8±20.3	75.4±14.6^e^
Index, mean ± SD	0.644±0.145	0.767±0.131^c^	0.780±0.123^c^	0.630±0.132	0.604±0.144	0.761±0.087^c^
DQOL, n		83			58	
Satisfaction, mean ± SD	3.0±0.7	2.2±0.8^c^	2.0±0.8^c^	3.0±0.8	3.2±0.7^d^	2.2±0.8^c^
Impact, mean ± SD	2.5±0.7	1.9±0.7^c^	1.8±0.6^c^	2.7±0.7	2.7±0.6	1.9±0.5^c^
Worry: social/vocational, mean ± SD	1.7±0.7	1.4±0.6^c^	1.4±0.6^c^	1.7±0.6	1.7±0.7	1.3±0.4^c^
Worry: diabetes-related, mean ± SD	2.1±0.8	1.8±0.8^e^	1.6±0.7^c^	2.3±0.8	2.4±0.9^d^	1.8±0.7^c^
Total, mean ± SD	2.5±0.6	1.9±0.6^c^	1.8±0.6^c^	2.6±0.7	2.7±0.6	1.9±0.5^c^
CGIC, n		80			57	
Better, great deal better (%)	-	72%	79%	-	0%	77%
Little, somewhat, moderately better (%)	-	27%	20%	-	5%	19%
No change, almost the same (%)	-	1%	1%	-	95%	4%
PGIC, n		84			58	
Better, great deal better (%)	-	67%	73%	-	0%	71%
Little, somewhat, moderately better (%)	-	32%	23%	-	7%	28%
No change, almost the same (%)	-	1%	5%	-	93%	2%

a CGIC, Clinician Global Impression of Change; CMM, conventional medical management; DN4, Douleur Neuropathique; DQOL, Diabetes Quality of Life; EQ-5D-5L, EuroQol 5-dimensional questionnaire; HbA1c, hemoglobin A1c; PGIC, Patient Global Impression of Change; SCS, spinal cord stimulation; SF-MPQ-2: Short-Form McGill Pain Questionnaire version 2; VAS, visual analog scale.

b Outcomes assessed in the per-protocol population at baseline and at 6 and 12 months for participants randomized to 10-kHz SCS+CMM and to CMM who crossed over after 6 months. HbA1c: laboratory assessment of percentage glycated hemoglobin; lower limb pain: participants reported pain intensity using a 10-cm VAS, where 0 indicates “no pain” and 10 indicates “worst pain imaginable,” responders are defined as participants with at least 50% pain relief from the baseline; the DN4 is a validated neuropathic pain measure, and a score of 4 or greater is consistent with a clinical diagnosis of painful diabetic neuropathy; the SF-MPQ-2 is a patient-reported outcome rating the intensity of 22 pain descriptors on a scale of 0 (none) to 10 (worst possible) and categorized into 4 subscales: continuous pain, intermittent pain, neuropathic pain, and affective component; neurologic assessment included motor strength and reflex testing, as well as sensory testing for light touch, pinprick, and 10-g monofilament. All follow-up assessments were compared with baseline, and the investigator categorized motor, reflex, and sensory separately as an “improvement,” “no change,” or a “deficit.” Overall neurologic improvement is defined as an improvement of motor, reflex, or sensory, without a deficit in any; the EQ-5D-5L questionnaire measures health-related quality of life, with an overall health VAS from 0 (the worst health you can imagine) to 100 (the best health you can imagine) and 5 items each with 5 levels of response that are converted to an index value on the basis of US population norms, ranging from −0.109 (a state worse than death) to 1.000 (perfect health); the DQOL measure is a 46-item patient questionnaire that assesses how diabetes impacts one’s life and can be divided into 4 subscales: satisfaction, impact, worry about social and vocational issues, and diabetes-related worry; the CGIC and PGIC ask the clinician and patient, respectively, to evaluate change since baseline in activity limitations, symptoms, emotions, and overall quality of life on a 7-point Likert scale.

c *P*<.001.

d *P*<.05.

e *P*<.01.

The NNT for 50% pain relief with 10-kHz SCS was 1.3 (95% CI, 1.1-1.4) on the basis of the proportion of pain responders among original treatment assignments at 6 months (74 of 87 in the 10-kHz SCS+CMM group vs 5 of 93 in the control arm).

At 6 months, 43 of 73 (59%) participants in the 10-kHz SCS+CMM group demonstrated improved neurologic function on examination, and this result was maintained through 12 months (52 of 76, 68%, Table 2). Similarly, for crossover patients after implant, 32 of 52 (62%) participants were observed to have improvement at 12 months. Nearly all with improved overall neurologic function had improvements on the sensory portion of their examination.

### Health-related Quality of Life

The participants in the 10-kHz SCS+CMM group rated their baseline overall health 58.7 of 100 (95% CI, 54.7-62.7) on the EQ-5D-5L VAS (Table 2). This improved by 14.7 points (95% CI, 10.3-19.0) at 6 months and by 17.0 points (95% CI, 11.9-22.0) at 12 months. The mean EQ-5D-5L index value was 0.644 of 1.0 (95% CI, 0.613-0.675) at baseline, which improved by 0.124 (95% CI, 0.090-0.157) at 6 months and by 0.136 (95% CI, 0.104-0.169) at 12 months (Figure 2B). The participants in the crossover group reported a mean EQ-5D-5L overall health VAS of 58.1 (95% CI, 52.6-63.6) at baseline, which improved by 17.3 points (95% CI, 11.2-23.4) at 12 months (Table 2). The mean EQ-5D-5L index score was 0.630 (95% CI, 0.596-0.665) at baseline and improved by 0.130 (95% CI, 0.094-0.166; Figure 2B).

The mean total Diabetes Quality of Life score at baseline was 2.5 of 5 (95% CI, 2.4-2.7) for the 10-kHz SCS+CMM group participants. The patients reported improvements across all 4 subscales by 6 months that were sustained through 12 months (Table 2). The crossover group had a baseline total score of 2.6 (95% CI, 2.5-2.8), which improved across all 4 subscales at 12 months (Table 2).

### Other Outcomes

Poor sleep quality was evidenced by baseline scores on the Pain and Sleep Questionnaire 3-Item Index of 5.9 of 10 (95% CI, 5.4-6.5) for the 10-kHz SCS+CMM group and 6.7 (95% CI, 6.1-7.2) for the crossover group (Figure 2C). Treatment with 10-kHz SCS significantly reduced pain interference with sleep, resulting in 62.4% (95% CI, 54.5-70.3) improvement for the original SCS group and 60.6% (95% CI, 52.3-69.0) improvement for the crossover group at 12 months.

Pain interference with daily living as measured by the Brief Pain Inventory for Painful Diabetic Peripheral Neuropathy was 6.2 of 10 (95% CI, 5.8-6.7) for the 10-kHz SCS+CMM group and 5.9 (95% CI, 5.4-6.4) for the crossover group (Figure 2D). Treatment with 10-kHz SCS significantly alleviated this interference.

Clinicians rated patients’ social, occupational, and psychological functioning by Global Assessment of Functioning, reporting mean baseline score of 63.1 of 100 (95% CI, 59.5-66.7) that improved by 18.4 points (95% CI, 14.4-22.4) at 6 months and 20.7 points (95% CI, 16.5-24.9) at 12 months for the 10-kHz SCS+CMM group (Figure 2E). For the crossover group, the mean baseline score of 62.1 (95% CI, 58.1-66.0) decreased by 4.9 points (95% CI, 1.7-8.1) at 6 months with continued CMM and increased by 16.9 points postimplant (95% CI, 12.4-21.4).

Participants reported high satisfaction with 10-kHz SCS at 12 months: 46 of 142 (46%) were “satisfied” and 85 of 142 (60%) were “very satisfied” among all implanted patients (Figure 2F). Both clinicians and participants reported that most were “better” or “a great deal better” compared with baseline on Global Impression of Change when treated with 10-kHz SCS (Table 2).

### Safety

In total, 154 participants received a permanent 10-kHz SCS device implant. The most common study-related adverse event was surgical site infection (n=8, 5.2%): 3 infections resolved and patients continued, whereas 5 (3.2%) patients required surgical explant, with 4 patients discontinuing and 1 later reimplanted and remaining in the study. There were neither stimulation-related neurologic deficits nor any explants for loss of efficacy. Two (1.3%) participants underwent surgical revision of the IPG location and lead migration for 1 (0.6%) participant required a revision procedure, but all 3 continued in the study.

Several participants discontinued because of health reasons unrelated to the study, consistent with the presence of significant comorbidities; however, retention was good among patients in the 10-kHz SCS group; of participants medically able to proceed with trial SCS, 154 of 181 (85%) received permanent device implants, and 148 of 154 (96%) of those remained in the study at 12 months.

## Discussion

This is the largest RCT to date for SCS treatment of PDN. Compared with pharmacotherapy and low-frequency SCS, 10-kHz SCS resulted in more profound pain relief and pronounced HRQoL improvements. Additionally, 10-kHz SCS does not induce continuous paresthesias as is required by conventional SCS, an advantage for patients with PDN who frequently already suffer uncomfortable paresthesia because of diabetic neuropathy.^43^ There were also observed neurologic improvements that, to the best of our knowledge, have not been reported previously for any PDN treatments.

A lack of sensation in the feet of patients with PDN limits activities of daily living and can result in debilitating sequelae, including injury from falling, fractures, foot ulceration, and lower limb amputation. Over half of the patients treated with 10-kHz SCS had notable improvement on neurologic examination, particularly improved sensation, and this was durable over 12 months. This potentially disease-modifying effect could have tremendous benefit for the safety and quality of life of patients with PDN and merits further research.

The estimates for the minimally important difference in EQ-5D-5L index specifically for individuals with type 2 diabetes range from 0.03 to 0.05.^44^ The differences observed in this study, 0.136 in the original 10-kHz SCS+CMM group and 0.130 in the crossover group, are 2.6 to 4.5 times the minimally important difference, consistent with substantial improvement in HRQoL.

There have been 2 prior RCTs evaluating SCS for PDN, both requiring continuous-induced paresthesias in the painful area using low-frequency SCS.^17^, ^18^, ^19^ Slangen et al^18^ randomized 36 patients, 3:2 to low-frequency SCS+CMM or CMM and reported results at 6 months. Additional follow-up was then reported in van Beek et al^19^, and the proportion of pain responders at 12 months was 6 of 16 (38%) during the day and 9 of 16 (56%) during the night. The participants reported improved EQ-5D-5L index scores over 12 months but no change in overall health VAS. Seventeen participants received a permanent low-frequency SCS device implant, 4 (23.5%) required surgical revision of lead placement, 2 (11.8%) required IPG replacements, and 1 (5.9%) required explantation because of infection. de Vos et al^17^ randomized 60 patients 2:1 to low-frequency SCS+CMM or CMM and reported results for up to 6 months. The proportion of pain responders among patients who completed the 6-month follow-up was 69% (25 of 36). The participants reported improved EQ-5D-5L overall health VAS, and 80% were satisfied with the treatment. In total, 37 participants received a permanent device implant, 1 (2.7%) participant developed an infection, and 3 (8.1%) participants required surgical revision of the leads or IPG over 6 months.

The effectiveness of low-frequency SCS attenuates over time.^45^^,^^46^ van Beek et al^19^ reported a similar trend of rising pain scores and decreasing responder rates by 12 months, continuing to 24 months, and saw no difference from baseline in EQ-5D-5L, well-being, depression, or sleep quantity at 24 months.^19^ In contrast, results with 10-kHz SCS are remarkably durable over 12 months, with no significant change in pain relief or HRQoL improvements, reflecting that the mechanism of action for 10-kHz SCS differs from low-frequency SCS.^22^^,^^23^

Procedure-related complications observed in this study aligned with expected SCS complication rates.^47^ The 5.2% incidence of infections is within the range of 2.5% to 10.0% (mean, 4.9%; 95% CI, 3.4-6.4) reported across SCS studies, including several cohorts without diabetes. Additionally, the incidence of lead migration, IPG and lead placement revision, and explants were all low compared with reports for SCS.^47^^,^^48^ These results imply that patients with PDN can be safely treated with 10-kHz SCS.

Patients with PDN medically appropriate for implant procedures have a high likelihood of success with 10-kHz SCS. Finnerup et al^12^ completed a systematic review and meta-analysis of RCTs for neuropathic pain medications vs placebo reporting NNTs for 50% pain relief. The NNT for 10-kHz SCS was 1.3, an improvement compared with pharmacotherapies (Table 3).

**Table 3:** Number Needed to Treat for PDN Treatments

PDN treatment	Number needed to treat^b^ (95% CI)
High-concentration (8%) capsaicin patches	10.6 (7.4-19)
Gabapentin, extended-release	8.3 (6.2-13.0)
Pregabalin	7.7 (6.5-9.4)
Serotonin-norepinephrine reuptake inhibitors	6.4 (5.2-8.4)
Gabapentin	6.3 (5.0-8.3)
Weak opioid agonists	4.7 (3.6-6.7)
Strong opioid agonists	4.3 (3.4-5.8)
Tricyclic antidepressants	3.6 (3.0-4.4)
10-kHz SCS^a^	1.3 (1.1-1.4)

a SCS, spinal cord stimulation.

b The number needed to treat represents the number of patients that need to be treated with an intervention to achieve 1 more responder with at least 50% pain relief compared with the control intervention. Finnerup et al^12^ completed a systematic review and meta-analysis of randomized controlled trials for neuropathic pain medications vs placebo. The current study results were used to calculate the number needed to treat for 10-kHz SCS compared with continued conventional medical management.

Long-term controlled pharmacotherapy studies are lacking; few extend beyond 12 weeks. Nonetheless, initial pharmacological management of PDN is appropriate and supported by guidelines.^8^ Those with refractory symptoms or intolerable adverse effects to pharmacotherapy should be considered for 10-kHz SCS, perhaps before the introduction of opioids, which carry potentially hazardous associated risks and lack data supporting long-term efficacy.^49^ Notably, the participants enrolled in our study had failed first-line treatments and many of the recommended second- and third-line PDN treatments, yet had substantial improvements with 10-kHz SCS.

The optional crossover study design provided a unique data set wherein patients serve as their own controls. Pain, health, and HRQoL measures remained unchanged or deteriorated over 6 months of CMM. Results for crossover patients postimplant are similar to those originally assigned to 10-kHz SCS. The participants in this study represent a large patient population who has exhausted the best available medical treatments and need effective alternatives.

The results for PDN are similar to those reported with 10-kHz SCS treatment of other chronic pain conditions. There were 85% pain responders at 12 months, which aligns with prior prospective studies with 10-kHz SCS reporting the proportion of responders at 12 months for neck pain (85%-89%), pelvic pain (77%), upper limb pain (76%-95%), back pain (79%), leg pain (79%), chronic postsurgical pain (78%), abdominal pain (82%), and nonsurgical back pain (90%).^24^^,^^50^, ^51^, ^52^, ^53^, ^54^, ^55^

This study was unblinded, which may influence the participants and investigators. Steps to minimize bias included concealed treatment allocation and random sequence generation. The treatment groups were well-matched for baseline characteristics, missing data were unlikely to affect the outcomes, and prespecified end points have been reported.^27^

The detailed neurologic examination has been previously described.^27^^,^^28^ Although care was taken to standardize the examination, interassessor variability should be considered a limitation. Further studies with more objective metrics for neurologic testing are needed to better understand and substantiate these findings.

Comparing a surgical intervention to medical management has potential for greater placebo effect in the active treatment arm; however, stable results with 10-kHz SCS over 12 months mitigate concerns about the placebo response.

In conclusion, 10-kHz SCS provided substantial and durable pain relief, improved HRQoL, better sleep quality, and neurologic improvements over 12 months. These data should support the use of 10-kHz SCS for patients with PDN with symptoms refractory to conservative care.

## Potential Competing Interests

Dr Petersen has received consulting fees from Abbott, Medtronic, Neuros Medical, Nevro, Saluda, and Vertos and research support from Medtronic, Neuros Medical, Nevro, ReNeuron, and Saluda. Dr Scowcroft has received research support from Boston Scientific, Nevro, Saluda, and Vertiflex. Dr Brooks is an employee of Nevro Corp. Dr White has received consulting fees from Lilly and CALIBR. Dr Sills has received research support from Nevro. Dr Amirdelfan has received consulting fees from Nevro, Nalu, Saluda, Biotronik, and Medtronic and research support from Nevro, Biotronik, Vivex, Saluda, and SPR Therapeutics. Dr Guirguis has received consulting fees from Nevro, Saluda, Avanos and research support from Abbott, Saluda, Neuros, Nevro, Nalu, and Avanos. Dr Xu has received research support from Nevro, NIH K08 Grant CA228039. Dr Yu has received research support from Nevro. Dr Nairizi has received consulting fees from Flowonix and Nevro and research support from Nevro. Dr Patterson has received consulting fees from Abbott, AIS, Allergan, Amgen, and CornerLoc and research support from Abbott, Biotronik, Flowonix, Nevro, Nuvectra, and Vertiflex. Dr Mehta has received consulting fees from Nevro, Salix Pharma, Biodelivery Sciences, and Averitas and research support from Boston Scientific, Medtronic, and Nevro. Dr Sayed has received consulting fees from Abbott, Boston Scientific, Nevro, Vertos, Vertiflex and research support from Abbott, Biotronic, Nevro, Vertos, Vertiflex. Dr Lad has received consulting fees from Nevro. Dr Goree has received consulting fees from Stratus Medical and Abbott and research support from SPR Therapeutics and Mainstay Medical. Dr Argoff has received consulting fees from Nevro, Vertex, Lilly, Pfizer, and Teva and research support from Allergan, Amgen, DSI, Lilly, Novartis, and Teva. Dr Nasr has received consulting fees from Siemens Healthineers, Horizon Therapeutics, Nevro, Neurogastrx, and Exelixis. Dr Taylor has received consulting fees from Medtronic, Nevro, and Saluda. Dr Caraway is an employee of Nevro Corp. Dr Mekhail has received consulting fees from Boston Scientific, Sollis Therapeutics, Saluda Medical, Nevro, Abbott (formerly Spinal Modulation), Vertos Medical, Nuvectra, and Relievant Medsystems; is a Medical Monitor for Mainstay’s RESTORE clinical trial; and has received research support from Avanos “Halyard,” Mallinckrodt, Mesoblast, and Neuros Medical. Other authors report no competing interests.

## References

[1] (2016). Global report on diabetes. World Health Organization. https://www.who.int/publications/i/item/9789241565257.

[2] Saeedi P., Petersohn I., Salpea P. (2019). Global and regional diabetes prevalence estimates for 2019 and projections for 2030 and 2045: results from the International Diabetes Federation Diabetes Atlas, 9^th^ edition. Diabetes Res Clin Pract.

[3] Sloan G., Selvarajah D., Tesfaye S. (2021). Pathogenesis, diagnosis and clinical management of diabetic sensorimotor peripheral neuropathy. Nat Rev Endocrinol.

[4] Boulton A.J., Armstrong D.G., Albert S.F. (2008). Comprehensive foot examination and risk assessment: a report of the task force of the foot care interest group of the American Diabetes Association, with endorsement by the American Association of Clinical Endocrinologists. Diabetes Care.

[5] Rastogi A., Goyal G., Kesavan R. (2020). Long term outcomes after incident diabetic foot ulcer: multicenter large cohort prospective study (EDI-FOCUS investigators) epidemiology of diabetic foot complications study: epidemiology of diabetic foot complications study. Diabetes Res Clin Pract.

[6] Zelman D.C., Brandenburg N.A., Gore M. (2006). Sleep impairment in patients with painful diabetic peripheral neuropathy. Clin J Pain.

[7] Abbott C.A., Malik R.A., van Ross E.R., Kulkarni J., Boulton A.J. (2011). Prevalence and characteristics of painful diabetic neuropathy in a large community-based diabetic population in the U.K. Diabetes Care.

[8] Pop-Busui R., Boulton A.J., Feldman E.L. (2017). Diabetic neuropathy: a position statement by the American Diabetes Association. Diabetes Care.

[9] Sloan G., Alam U., Selvarajah D., Tesfaye S. (2022). The treatment of painful diabetic neuropathy. Curr Diabetes Rev.

[10] Ismail-Beigi F., Craven T., Banerji M.A. (2010). Effect of intensive treatment of hyperglycaemia on microvascular outcomes in type 2 diabetes: an analysis of the ACCORD randomised trial. Lancet.

[11] Bril V., England J., Franklin G.M. (2011). Evidence-based guideline: treatment of painful diabetic neuropathy: report of the American Academy of Neurology, the American Association of Neuromuscular and Electrodiagnostic Medicine, and the American Academy of Physical Medicine and Rehabilitation. Neurology.

[12] Finnerup N.B., Attal N., Haroutounian S. (2015). Pharmacotherapy for neuropathic pain in adults: a systematic review and meta-analysis. Lancet Neurol.

[13] Yang M., Qian C., Liu Y. (2015). Suboptimal treatment of diabetic peripheral neuropathic pain in the United States. Pain Med.

[14] Robinson-Papp J., Simpson D.M. (2007). Safety profile of treatment in diabetic peripheral neuropathic pain. Pain Med.

[15] Tesfaye S., Watt J., Benbow S.J., Pang K.A., Miles J., MacFarlane I.A. (1996). Electrical spinal-cord stimulation for painful diabetic peripheral neuropathy. Lancet.

[16] Pluijms W.A., Slangen R., Bakkers M. (2012). Pain relief and quality-of-life improvement after spinal cord stimulation in painful diabetic polyneuropathy: a pilot study. Br J Anaesth.

[17] de Vos C.C., Meier K., Zaalberg P.B. (2014). Spinal cord stimulation in patients with painful diabetic neuropathy: a multicentre randomized clinical trial. Pain.

[18] Slangen R., Schaper N.C., Faber C.G. (2014). Spinal cord stimulation and pain relief in painful diabetic peripheral neuropathy: a prospective two-center randomized controlled trial. Diabetes Care.

[19] van Beek M., Slangen R., Schaper N.C. (2015). Sustained treatment effect of spinal cord stimulation in painful diabetic peripheral neuropathy: 24-month follow-up of a prospective two-center randomized controlled trial. Diabetes Care.

[20] van Beek M., Geurts J.W., Slangen R. (2018). Severity of neuropathy is associated with long-term spinal cord stimulation outcome in painful diabetic peripheral neuropathy: five-year follow-up of a prospective two-center clinical trial. Diabetes Care.

[21] North R.B., Ewend M.G., Lawton M.T., Piantadosi S. (1991). Spinal cord stimulation for chronic, intractable pain: superiority of “multi-channel” devices. Pain.

[22] De Carolis G., Paroli M., Tollapi L. (2017). Paresthesia-independence: an assessment of technical factors related to 10 kHz paresthesia-free spinal cord stimulation. Pain Physician.

[23] Lee K.Y., Bae C., Lee D. (2020). Low-intensity, kilohertz frequency spinal cord stimulation differently affects excitatory and inhibitory neurons in the rodent superficial dorsal horn. Neuroscience.

[24] Kapural L., Yu C., Doust M.W. (2015). Novel 10-kHz high-frequency therapy (HF10 therapy) is superior to traditional low-frequency spinal cord stimulation for the treatment of chronic back and leg pain: the SENZA-RCT randomized controlled trial. Anesthesiology.

[25] Kapural L., Yu C., Doust M.W. (2016). Comparison of 10-kHz high-frequency and traditional low-frequency spinal cord stimulation for the treatment of chronic back and leg pain: 24-month results from a multicenter, randomized, controlled pivotal trial. Neurosurgery.

[26] Galan V., Scowcroft J., Chang P. (2020). 10-kHz spinal cord stimulation treatment for painful diabetic neuropathy: results from post-hoc analysis of the SENZA-PPN study. Pain Manag.

[27] Petersen E.A., Stauss T.G., Scowcroft J.A. (2021). Effect of high-frequency (10-kHz) spinal cord stimulation in patients with painful diabetic neuropathy: a randomized clinical trial. JAMA Neurol.

[28] Petersen E.A., Stauss T.G., Scowcroft J.A. (2022). Durability of high-frequency 10-kHz spinal cord stimulation for patients with painful diabetic neuropathy refractory to conventional treatments: 12-month results from a randomized controlled trial. Diabetes Care.

[29] Chen J.L., Hesseltine A.W., Nashi S.E. (2022). A real-world analysis of high-frequency 10 kHz spinal cord stimulation for the treatment of painful diabetic peripheral neuropathy. J Diabetes Sci Technol.

[30] Mekhail N.A., Argoff C.E., Taylor R.S. (2020). High-frequency spinal cord stimulation at 10 kHz for the treatment of painful diabetic neuropathy: design of a multicenter, randomized controlled trial (SENZA-PDN). Trials.

[31] Williams H.C., Burden-Teh E., Nunn A.J. (2015). What is a pragmatic clinical trial?. J Invest Dermatol.

[32] McCormack H.M., Horne D.J., Sheather S. (1988). Clinical applications of visual analogue scales: a critical review. Psychol Med.

[33] Bouhassira D., Attal N., Alchaar H. (2005). Comparison of pain syndromes associated with nervous or somatic lesions and development of a new neuropathic pain diagnostic questionnaire (DN4). Pain.

[34] Dworkin R.H., Turk D.C., Revicki D.A. (2009). Development and initial validation of an expanded and revised version of the Short-form McGill Pain Questionnaire (SF-MPQ-2). Pain.

[35] Zelman D.C., Gore M., Dukes E., Tai K.S., Brandenburg N. (2005). Validation of a modified version of the Brief Pain Inventory for Painful Diabetic Peripheral Neuropathy. J Pain Symptom Manage.

[36] Herdman M., Gudex C., Lloyd A. (2011). Development and preliminary testing of the new five-level version of EQ-5D (EQ-5D-5L). Qual Life Res.

[37] (1988). Reliability and validity of a diabetes quality-of-life measure for the diabetes control and complications trial (DCCT). The DCCT research group. Diabetes Care.

[38] Ayearst L., Harsanyi Z., Michalko K.J. (2012). The Pain and Sleep Questionnaire three-item index (PSQ-3): a reliable and valid measure of the impact of pain on sleep in chronic nonmalignant pain of various etiologies. Pain Res Manag.

[39] American Psychiatric Association (2000).

[40] Booth J., Young M.J. (2000). Differences in the performance of commercially available 10-g monofilaments. Diabetes Care.

[41] England J.D., Gronseth G.S., Franklin G. (2005). Distal symmetrical polyneuropathy: a definition for clinical research. A report of the American Academy of Neurology, the American Association of Electrodiagnostic Medicine, and the American Academy of Physical Medicine and Rehabilitation. Arch Phys Med Rehabil.

[42] Spallone V., Morganti R., D’Amato C., Greco C., Cacciotti L., Marfia G.A. (2012). Validation of DN4 as a screening tool for neuropathic pain in painful diabetic polyneuropathy. Diabet Med.

[43] Strand N.H., Burkey A.R. (2022). Neuromodulation in the treatment of painful diabetic neuropathy: a review of evidence for spinal cord stimulation. J Diabetes Sci Technol.

[44] McClure N.S., Sayah F.A., Ohinmaa A., Johnson J.A. (2018). Minimally important difference of the EQ-5D-5L index score in adults with type 2 diabetes. Value Health.

[45] Hayek S.M., Veizi E., Hanes M. (2015). Treatment-limiting complications of percutaneous spinal cord stimulator implants: a review of eight years of experience from an academic center database. Neuromodulation.

[46] Fishman M.A., Antony A., Esposito M., Deer T., Levy R. (2019). The evolution of neuromodulation in the treatment of chronic pain: forward-looking perspectives. Pain Med.

[47] Eldabe S., Buchser E., Duarte R.V. (2016). Complications of spinal cord stimulation and peripheral nerve stimulation techniques: a review of the literature. Pain Med.

[48] Van Buyten J.P., Wille F., Smet I. (2017). Therapy-related explants after spinal cord stimulation: results of an international retrospective chart review study. Neuromodulation.

[49] Gupta M., Knezevic N.N., Abd-Elsayed A., Ray M., Patel K., Chowdhury B. (2021). Treatment of painful diabetic neuropathy-a narrative review of pharmacological and interventional approaches. Biomedicines.

[50] Verrills P., Salmon J., Russo M., Gliner B., Barnard A., Caraway D. (2020). 10 kHz spinal cord stimulation for chronic upper limb and neck pain: Australian experience. Eur Spine J.

[51] Amirdelfan K., Vallejo R., Benyamin R. (2020). High-frequency spinal cord stimulation at 10 kHz for the treatment of combined neck and arm pain: results from a prospective multicenter study. Neurosurgery.

[52] Tate J.L., Stauss T., Li S., Rotte A., Subbaroyan J. (2021). A prospective, multi-center, clinical trial of a 10-kHz spinal cord stimulation system in the treatment of chronic pelvic pain. Pain Pract.

[53] Gupta M., Scowcroft J., Kloster D. (2020). 10-kHz spinal cord stimulation for chronic postsurgical pain: results from a 12-month prospective, multicenter study. Pain Pract.

[54] Kapural L., Gupta M., Paicius R. (2020). Treatment of chronic abdominal pain with 10-kHz spinal cord stimulation: safety and efficacy results from a 12-month prospective, multicenter, feasibility study. Clin Transl Gastroenterol.

[55] Al-Kaisy A., Palmisani S., Smith T.E. (2017). 10 kHz high-frequency spinal cord stimulation for chronic axial low back pain in patients with no history of spinal surgery: a preliminary, prospective, open label and proof-of-concept study. Neuromodulation.

