# Supplementary material for: High-Frequency 10-kHz Spinal Cord Stimulation Improves Health-Related Quality of Life in Patients With Refractory Painful Diabetic Neuropathy: 12-Month Results From a Randomized Controlled Trial
Source: Mayo Clin Proc Innov Qual Outcomes. 2022 Jul 1;6(4):347–60. doi: 10.1016/j.mayocpiqo.2022.05.003 (PMC9256824; doi:10.1016/j.mayocpiqo.2022.05.003)
Supplement: Supplemental Data [file mmc1.pdf]

Supplemental Figure 1: Pain results for CMM continuers in per-protocol population over 12 months

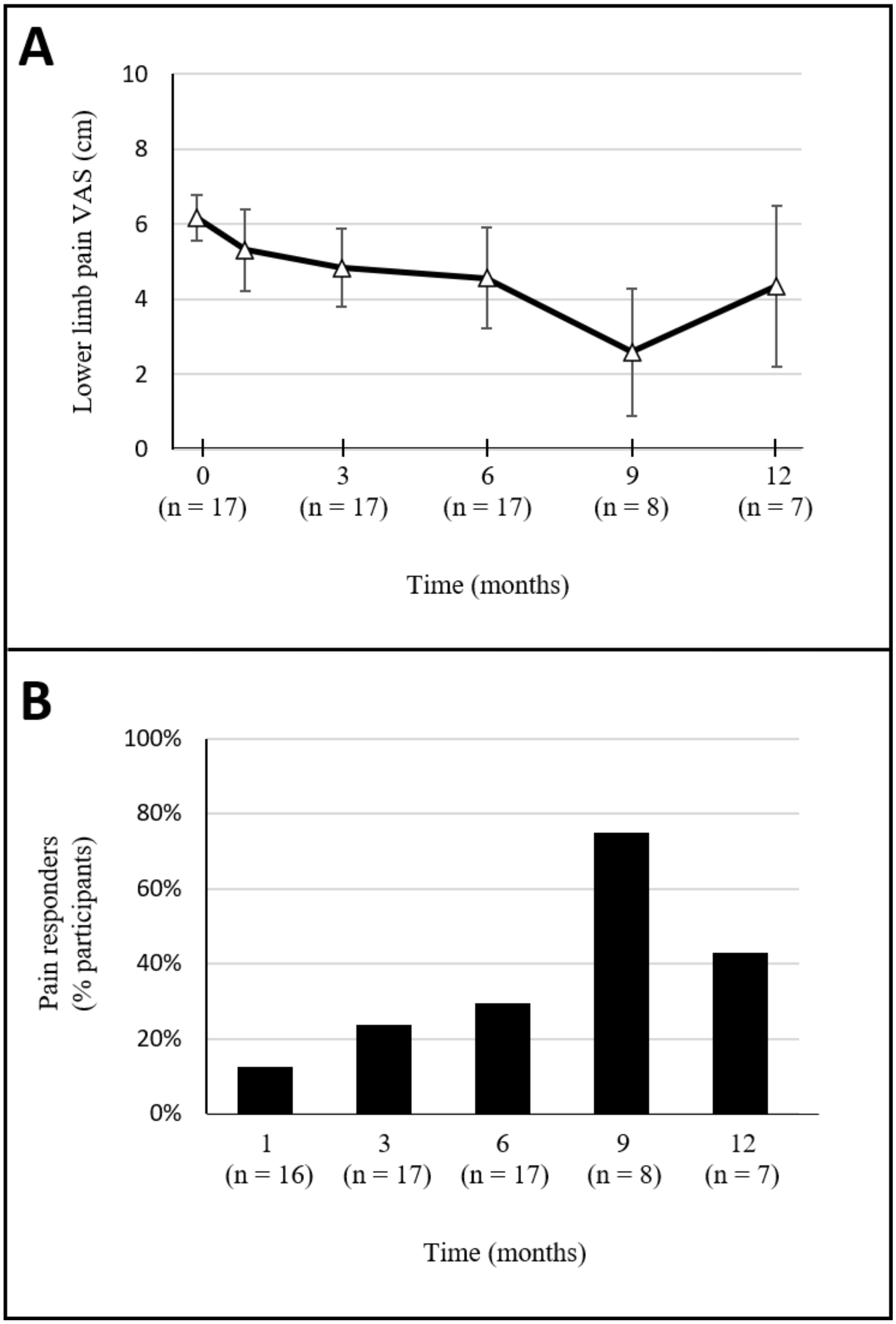

Supplemental Figure 1: A) Average lower limb pain scores over 12 months for CMM participants who remained in CMM. The number of patients who completed each visit shown in parentheses. Error bars: 95% CI. **B)** Proportion of CMM continuers who were pain responders, defined as those with at least 50% pain relief from baseline, at 1, 3, 6, 9, and 12 months. The number of patients who completed each visit shown in parentheses.

Supplemental Table 1: **Outcomes for CMM continuers in per-protocol population**

	Outcome Measure	Baseline	3 Months	6 Months	12 Months
Patient #1	Pain VAS (cm)	6.65	0	0	8.05
	Pain relief (%)	-	100	100	-21
	DN4	1	0	1	1
	EQ-5D-5L Health VAS	70	100	100	70
	EQ-5D-5L Index	0.777	0.844	0.550	0.820
	DQOL total	1.3	1.3	1.3	1.2
	GAF	61	61	61	61
Patient #2	Pain VAS (cm)	6.55	3.2	3.9	3.95
	Pain relief (%)	-	51	40	40
	DN4	8	6	6	6
	EQ-5D-5L Health VAS	55	50	40	60
	EQ-5D-5L Index	0.714	0.719	0.771	0.796
	DQOL total	2.4	2.4	2.4	2.2
	GAF	55	55	65	65
Patient #3	Pain VAS (cm)	7.0	8.25	7.2	DNC
	Pain relief (%)	-	-18	-3	-
	DN4	7	5	5	DNC
	EQ-5D-5L Health VAS	70	60	80	DNC
	EQ-5D-5L Index	0.732	0.612	0.741	DNC
	DQOL total	1.6	2.0	1.7	DNC
	GAF	80	80	80	DNC
Patient #4	Pain VAS (cm)	7.05	6.05	7.1	DNC
	Pain relief (%)	-	14	-1	-
	DN4	6	6	6	DNC
	EQ-5D-5L Health VAS	65	65	30	DNC
	EQ-5D-5L Index	0.669	0.741	0.650	DNC
	DQOL total	1.7	2.1	2.6	DNC
	GAF	81	81	81	DNC
Patient #5	Pain VAS (cm)	2.7	5.3	7.6	DNC
	Pain relief (%)	-	-96	-181	-
	DN4	6	7	8	DNC
	EQ-5D-5L Health VAS	65	65	35	DNC
	EQ-5D-5L Index	0.741	0.735	0.584	DNC
	DQOL total	2.1	2.1	3.2	DNC
	GAF	75	75	75	DNC
Patient #6	Pain VAS (cm)	6.45	3.65	0.25	1.5
	Pain relief (%)	-	43	96	77
	DN4	6	6	5	5
	EQ-5D-5L Health VAS	35	25	60	60
	EQ-5D-5L Index	0.616	0.565	0.706	0.689
	DQOL total	2.5	2.0	1.7	1.6
	GAF	65	65	80	65
Patient #7	Pain VAS (cm)	6.0	5.7	7.2	DNC
	Pain relief (%)	-	5	-20	-
	DN4	5	3	2	DNC
	EQ-5D-5L Health VAS	100	70	80	DNC
	EQ-5D-5L Index	0.845	0.790	0.655	DNC
	DQOL total	1.6	1.9	2.1	DNC
	GAF	85	85	92	DNC

	Outcome Measure	Baseline	3 Months	6 Months	12 Months
Patient #8	Pain VAS (cm)	3.65	5.3	5.45	DNC
	Pain relief (%)	-	-45	-49	-
	DN4	6	5	7	DNC
	EQ-5D-5L Health VAS	95	80	25	DNC
	EQ-5D-5L Index	0.826	0.723	0.813	DNC
	DQOL total	1.7	1.7	1.8	DNC
	GAF	90	65	65	DNC
Patient #9	Pain VAS (cm)	6.0	6.1	6.75	DNC
	Pain relief (%)	-	-2	-12	-
	DN4	8	6	8	DNC
	EQ-5D-5L Health VAS	40	45	70	DNC
	EQ-5D-5L Index	0.818	0.827	0.827	DNC
	DQOL total	2.9	2.4	2.4	DNC
	GAF	55	54	61	DNC
Patient #10	Pain VAS (cm)	7.15	5.4	0.65	1.2
	Pain relief (%)	-	24	91	83
	DN4	6	7	6	8
	EQ-5D-5L Health VAS	85	75	85	85
	EQ-5D-5L Index	0.741	0.806	0.861	1.000
	DQOL total	2.1	1.9	1.8	1.5
	GAF	65	65	81	90
Patient #11	Pain VAS (cm)	6.55	5.65	5.95	DNC
	Pain relief (%)	-	14	9	-
	DN4	6	6	6	DNC
	EQ-5D-5L Health VAS	35	70	44	DNC
	EQ-5D-5L Index	0.719	0.732	0.719	DNC
	DQOL total	2.5	2.1	2.4	DNC
	GAF	39	45	45	DNC
Patient #12	Pain VAS (cm)	6.48	7.2	7.45	7.65
	Pain relief (%)	-	-11	-15	-18
	DN4	8	7	8	8
	EQ-5D-5L Health VAS	80	25	85	60
	EQ-5D-5L Index	0.650	0.598	0.626	0.645
	DQOL total	1.9	2.3	2.0	2.4
	GAF	61	61	65	42
Patient #13	Pain VAS (cm)	8.02	7.5	6.8	DNC
	Pain relief (%)	-	6	15	-
	DN4	6	4	7	DNC
	EQ-5D-5L Health VAS	20	90	15	DNC
	EQ-5D-5L Index	0.591	0.389	0.389	DNC
	DQOL total	2.5	4.0	3.3	DNC
	GAF	39	40	55	DNC
Patient #14	Pain VAS (cm)	5.7	2.85	1.55	2.15
	Pain relief (%)	-	50	73	62
	DN4	6	5	5	5
	EQ-5D-5L Health VAS	80	75	75	75
	EQ-5D-5L Index	0.827	0.827	0.806	0.827
	DQOL total	1.8	1.8	1.8	1.8
	GAF	83	80	80	55

	Outcome Measure	Baseline	3 Months	6 Months	12 Months
Patient #15	Pain VAS (cm)	7.05	0.9	3.6	DNC
	Pain relief (%)	-	87	49	-
	DN4	10	5	5	DNC
	EQ-5D-5L Health VAS	50	60	60	DNC
	EQ-5D-5L Index	0.460	0.708	0.530	DNC
	DQOL total	2.0	1.8	1.7	DNC
	GAF	87	55	45	DNC
Patient #16	Pain VAS (cm)	6.4	4.2	1.2	5.9
	Pain relief (%)	-	34	81	8
	DN4	8	3	6	6
	EQ-5D-5L Health VAS	72.5	60	25	22
	EQ-5D-5L Index	0.506	0.710	0.499	0.258
	DQOL total	2.7	2.5	2.3	2.1
	GAF	58	69	48	43
Patient #17	Pain VAS (cm)	5.6	4.9	4.9	DNC
	Pain relief (%)	-	12	12	-
	DN4	7	2	DNC	DNC
	EQ-5D-5L Health VAS	60	45	65	DNC
	EQ-5D-5L Index	0.810	0.790	0.826	DNC
	DQOL total	1.7	1.7	1.6	DNC
	GAF	54	66	DNC	DNC

DNC: did not complete

Supplemental Table 2: **Outcomes for participants excluded from per-protocol population**

	Treatment Assigned<br>Treatment Received	Outcome Measure	Baseline	3 Months	6 Months	12 Months
Patient #1	CMM<br><br>CMM	Pain VAS (cm)	4.8	DNC	1.05	3.2
		Pain relief (%)	-	-	92	33
		DN4	8	DNC	5	5
		EQ-5D-5L Health VAS	55	DNC	30	45
		EQ-5D-5L Index	0.708	DNC	0.613	0.725
		DQOL total	3.2	DNC	3.0	2.7
		GAF	65	DNC	85	85
Patient #2	CMM<br><br>10 kHz SCS after 6 months	Pain VAS (cm)	8.15	DNC	9.6	0
		Pain relief (%)	-	-	-18	100
		DN4	8	DNC	8	4
		EQ-5D-5L Health VAS	DNC	DNC	5	100
		EQ-5D-5L Index	DNC	DNC	0.371	1.000
		DQOL total	3.8	DNC	4.0	1.1
		GAF	40	DNC	80	100
Patient #3	10 kHz SCS<br><br>10 kHz SCS	Pain VAS (cm)	5.55	DNC	0.85	0.3
		Pain relief (%)	-	-	85	95
		DN4	4	DNC	2	4
		EQ-5D-5L Health VAS	45	DNC	80	90
		EQ-5D-5L Index	0.714	DNC	0.880	0.826
		DQOL total	2.2	DNC	1.5	1.7
		GAF	51	DNC	80	92

DNC: did not complete
